# Exploring young adult cancer survivors’ perspectives on generative AI chatbots for symptom support

**DOI:** 10.1007/s00520-026-11254-0

**Published:** 2026-09-23

**Authors:** Yupawadee Kantabanlang, Grace Kanzawa-Lee, Rachel A. Pozzar, Robert Knoerl

**Affiliations:** 1 Department of Human Behavior and Clinical Sciences, University of Michigan School of Nursing, Ann Arbor, MI 48109 USA; 2 Phyllis F. Cantor Center for Research in Nursing and Patient Care Services, Dana-Farber Cancer Institute, Boston, MA 02215 USA; 3 School of Nursing, Bouvé College of Health Sciences, Northeastern University, Boston, MA 02115 USA; 4 Department of Health Behavior and Clinical Sciences, University of Michigan School of Nursing, 400 North Ingalls St., Office 2350, Ann Arbor, MI 48109 USA

**Keywords:** Generative artificial intelligence, Young adult, Cancer treatment-related symptoms, Qualitative research

## Abstract

**Background:**

Young adult cancer survivors (ages 18–39) often experience persistent treatment-related symptoms that negatively affect daily functioning, psychosocial well-being, and quality of life, yet report unmet educational needs for symptom management. Generative artificial intelligence (AI) technologies, such as large language model-powered chatbots, offer scalable, personalized, and empathetic symptom support but remain largely untested in young adults’ survivorship care.

**Objective:**

This qualitative descriptive study explored young adult cancer survivors’ perspectives, preferences, and experiences related to the use of generative AI chatbots for oncology symptom management.

**Methods:**

Eighteen post-treatment young adult cancer survivors who had completed cancer treatment within the past two years were recruited from the University of Michigan Rogel Cancer Center. Participants completed the PROMIS-29, PROMIS Self-Efficacy for Managing Symptoms, and the Digital Health Literacy Scale to quantify symptom burden, self-efficacy, and digital readiness. Semi-structured interviews explored experiences with cancer treatment-related symptoms and AI use, including preferences for personalization, tone, functionality, and data governance.

**Results:**

Qualitative analyses revealed three themes: (1) the need for personalized, emotionally supportive, and credible AI symptom guidance; (2) accessible and user-centered design drives engagement with AI symptom support; and (3) trust, privacy, and human oversight as preconditions for AI symptom support. Survivors viewed AI as a supplemental resource to enhance symptom monitoring, decision support, and emotional reassurance without replacing clinical care.

**Conclusion:**

Generative AI chatbots were perceived as acceptable and potentially valuable for AYA symptom management. Findings can guide the development of tailored digital self-management interventions responsive to the complex and evolving needs of this population.

**Supplementary Information:**

The online version contains supplementary material available at https://doi.org/10.1007/s00520-026-11254-0.

## Introduction

Cancer treatment-related symptom experiences in post-treatment young adult cancer survivors are highly individualized and often insufficiently addressed in survivorship care [1]. Cancer treatment-related symptoms may continue long after the completion of active treatment and can substantially disrupt daily functioning, psychosocial well-being, and long-term quality of life during a critical developmental period characterized by transitions in education, employment, relationships, and identity formation [2, 3]. Despite this burden, many young adult survivors report unmet educational needs regarding the late effects of cancer treatment [4, 5].

Digital health technologies offer promising solutions to bridge these gaps by extending symptom support beyond traditional clinical encounters. AI-based conversational agents, or chatbots, in particular, have demonstrated potential to deliver symptom education, self-management guidance, triage support, and emotional reassurance in cancer care [6–9]. However, these tools are most often developed and evaluated in older adult cancer survivors [10], with minimal attention to developmentally distinct groups such as adolescent or young adult (AYA) cancer survivors, whose psychosocial contexts, technology use patterns, and survivorship challenges differ [11]. Specifically, there is a lack of empirical evidence examining how young adult cancer survivors perceive, trust, and wish to engage with generative AI chatbots for oncology symptom management.

This study sought to address this gap by exploring young adult cancer survivors’ preferences and experiences related to the use of generative AI chatbots for oncology symptom management. The primary aim was to identify the key factors that young adult cancer survivors consider most important when engaging with an AI-powered chatbot for symptom support, including usability, trust, personalization, accuracy, and integration with clinical care.

## Methods

### Design, sample, and setting

A qualitative descriptive design [12] was used to determine young adults’ preferences and experiences with AI-powered chatbots for oncology symptom management. Eighteen post-treatment young adult cancer survivors were recruited from the University of Michigan Rogel Cancer Center. Participants were eligible if they were 18–39 years of age, English speaking, and had completed cancer treatment at least one month but no more than 2 years prior to enrollment. Given the qualitative descriptive design of the study and evidence suggesting that AYAs may continue to experience physical symptoms and ongoing support or information needs for years following completion of treatment [13, 14], participants were eligible if they were at different points in the post-treatment period to capture a range of survivorship experiences and perspectives regarding the potential role of AI-powered chatbots for symptom support.

Participant consent was obtained through an electronic consent form at the beginning of a patient-reported outcomes survey. The consent page informed participants that clicking “next” to proceed to the survey indicated their consent to participate in the study. The study was reviewed by the University of Michigan Institutional Review Board and considered to be an exempt study (HUM00278198).

### Measures

#### Semi-structured interview guide

Interview topics (Table 1) included questions to identify young adults’ priority physical and psychological symptoms post-treatment, preferred language and tone for chatbot communication, and suggestions for escalation logic (i.e., when the chatbot should encourage users to contact providers). The interview guide did not include questions pertaining to the use of AI chatbots for specific cancer treatment-related symptoms as the goal of the interview was to broadly explore participants’ perspectives and preferences regarding AI-powered chatbots for symptom support.

**Table 1:** Semi-Structured Interview Guide

Questions
1. Tell me a bit about your cancer diagnosis and any symptoms you had due to your cancer treatment - *Probe: During or after treatment, which symptoms affected your daily life the most?* - *Probe: Are there any symptoms that you feel are often overlooked by healthcare providers?* - *Probe: What have you used to manage your cancer treatment-related symptoms? If yes, how did you learn about those treatments (e.g., provider, peer support group, social media, professional organization, online)*
2. How, if at all, have you used AI in your cancer treatment or to manage your symptoms? 3. How else do you imagine AI could help with your cancer treatment symptoms? - *Probe: What benefits would you expect from using a chatbot to help manage your symptoms? (e.g., support, guidance, fewer clinic visits)* - *Probe: What might make you more likely to use a chatbot during or after treatment?* - *Probe: What do you think are barriers or problems to using chatbots to manage symptoms?* - *Probe: Would you have any concerns about sharing certain types of information or information about certain symptoms with the chatbot?*
If a chatbot were helping you track or manage symptoms, what kind of tone or language would make you feel most comfortable? (e.g., friendly, professional, casual, motivational) - *Probe: Are there any types of language or expressions that would make you feel uncomfortable or not taken seriously?* - *Probe: What features would make the chatbot more helpful or supportive for someone your age? (e.g., communicate via voice or text or both, file or photo upload)* - *Probe: How would you like to access the AI chatbot to manage cancer treatment symptoms in the future? (e.g., app, website browser, etc.)*
4. In which situations would you want the chatbot to report a symptom or concern to a healthcare provider? - *Probe: Would you want a chatbot to automatically report concerning symptoms to a provider? Or give you advice but leave it up to you to choose whether to contact your provider? Please explain your reasoning.”* - *Probe: Do you think the chatbot should always ask your permission before alerting a provider or caregiver? Why or why not?* - *Probe: What concerns do you have about the chatbot deciding when to report a symptom or concern to a healthcare provider?*

##### Patient-reported outcomes measurement information system (PROMIS-29) v2.0

The PROMIS-29 consists of brief (four-item) subscales that measure physical function (transformed score range 22.9–56.9), anxiety (40.3–81.6), depression (41.0–79.4), fatigue (33.7–75.8), sleep disturbance (32.0–73.33), pain interference (41.6–75.6), and satisfaction with participation in social roles (29–64.1). The PROMIS-29 also contains a 0–10 measure of average pain intensity [15]. Higher scores on all symptom subscales represent worse symptom severity, while higher scores on the satisfaction with participation in social roles subscale represent higher satisfaction.

#### Digital health care literacy scale

The three-item Digital Health Literacy Scale measures participants’ self-reported confidence in independently using, setting up, and/or troubleshooting computer-related applications (e.g., programs and video conferencing). The items are scored from 0 to 4, with higher scores reflecting greater agreement with each statement [16].

#### PROMIS self-efficacy for managing symptoms

The PROMIS Self-Efficacy for Managing Chronic Conditions-Managing Symptoms–Short Form 8a measures ones’ confidence in managing symptoms associated with a chronic condition at various settings (e.g., home or public) and confidence to prevent such symptoms from interfering with common activities of daily living [15]. Transformed total scores range from 22.67 to 63.85 (i.e., higher scores represent greater self-efficacy). The measure has demonstrated strong concurrent validity with the Self-Efficacy for Managing Chronic Disease 6-Item scale and strong internal consistency reliability (Cronbach’s alpha > 0.90) [17].

### Data collection procedures

Participants were emailed a link to complete a demographic questionnaire and patient-reported outcome measures. Participants completed the electronic survey before the semi-structured interview. The survey data were used to describe (i.e., medians and ranges for continuous variables and frequencies and percentages for categorical variables) demographic, clinical, symptom, self-efficacy, and digital health literacy information of the study sample and did not inform the interview guide or qualitative analysis. After survey completion, participants were contacted via email to schedule a 15–30-min semi-structured interview, conducted via secure video conferencing. All interviews were facilitated by a post-doctoral nurse scientist and audio-recorded with participant permission. To protect confidentiality, participants were identified by first name only during interview sessions. Participants were asked to describe their symptom experiences and share their perspectives on using an AI-powered chatbot for symptom support. Upon completing the survey and interview, participants received a $50 gift card.

### Qualitative analyses

Qualitative data were analyzed using an inductive content analytic approach [18], allowing patterns and themes to emerge from participants’ accounts. The sample size was guided by thematic saturation rather than a predetermined numerical target [19]. The interviewer and principal investigator reviewed the findings from the interviews over time to assess whether new perspectives were emerging. After 18 interviews, no substantively new perspectives emerged, and the research team determined that thematic saturation had been reached.

Semi-structured interviews were transcribed verbatim by a professional transcription service and checked for accuracy by the interviewer. Transcripts were not returned to participants for member checking. All qualitative data were managed using NVivo version 15 (QSR International Pty Ltd) [20, 21]. Following a review of the transcribed interviews, an initial coding framework was developed by the interviewer and refined in collaboration with the principal investigator. The interviewer then applied the finalized codes across all transcripts. Data were subsequently summarized in a framework matrix at the participant level to facilitate comparison and synthesis (Supplementary Information). The broader study team reviewed the matrix to interpret patterns across cases and iteratively identify overarching themes.

## Results

### Sample characteristics

**Fig. 1:**
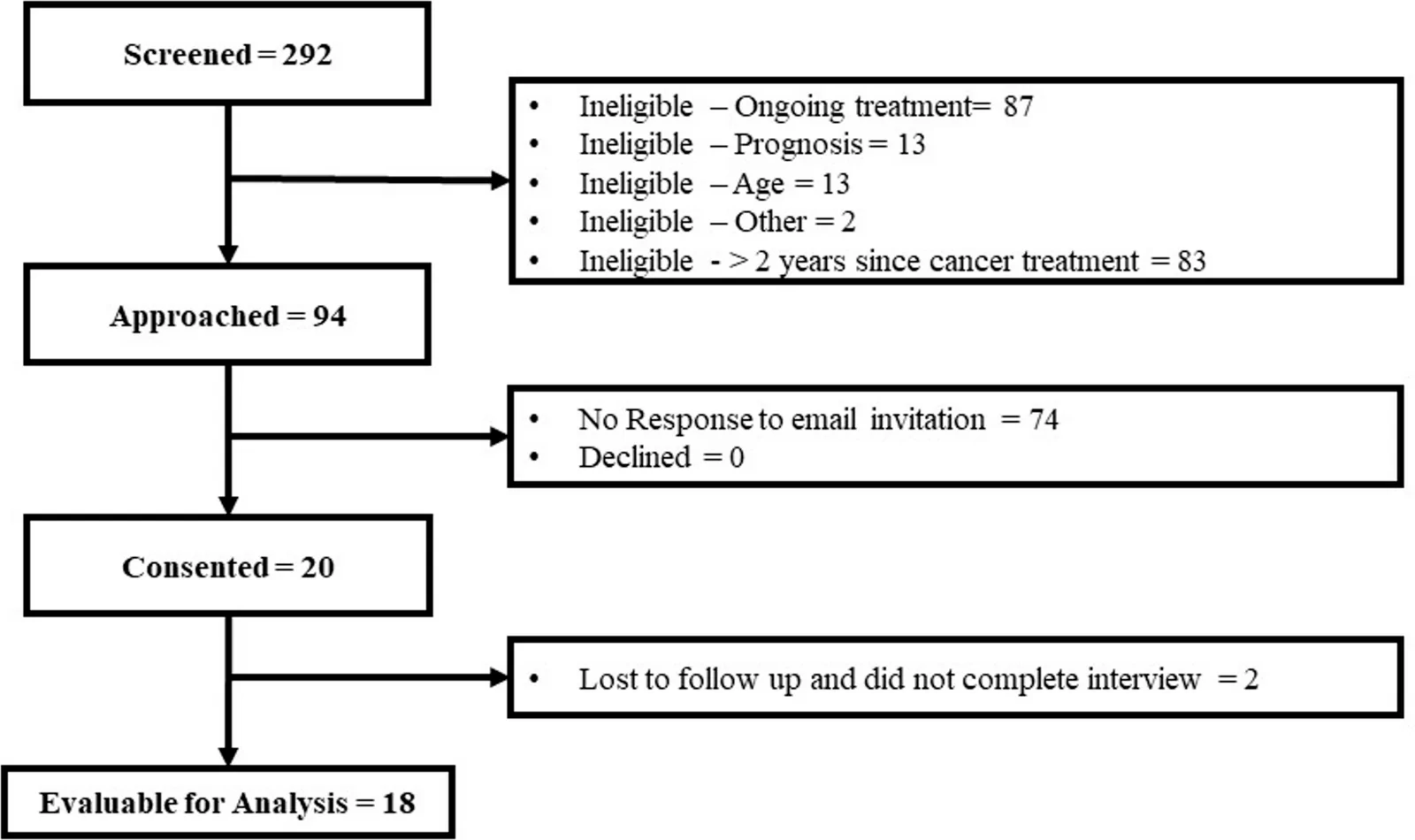
Participant recruitment and study flow. The figure describes participant flow through the study

Figure 1 describes participant flow through the study. Participant recruitment and data collection occurred between September and December 2025. Eighteen young adult cancer survivors completed the interview and survey. Table 2 describes participants’ baseline demographic and patient-reported symptom severity characteristics. The median age was 29 years (*range*: 18–39). Most participants identified as female (67%) and White (72%). Education levels ranged from high school diploma (11%) to post-graduate degree (28%). The most common cancer types were breast cancer (39%), lymphoma (28%), sarcoma or bone cancer (11%), and melanoma (11%). Participants had completed cancer treatment a median of 8.5 months ago (*range*: 1–18) at the time of study enrollment. Participants’ PROMIS anxiety severity was mild to moderate (median *T*-score: 59.55) [22], while the other PROMIS-29 symptom scores were near the US general population mean (score = 50) [23]. Median scores for all three Digital Health Care Literacy Scale items were 4.0 (range: 3–4), indicating high confidence in using digital applications, setting up video communication, and troubleshooting basic technical issues.

**Table 2:** Participant demographic, clinical characteristics, and baseline patient-reported outcome measures (*N* = 18)

Characteristic	Value
Age	
* Median* (*range*)	29 (18–39)
Sex	
Female	12 (67%)
Male	6 (33%)
Race	
Asian	3 (17%)
Black or African-American	1 (5.5%)
White	13 (72%)
Other	1 (5.5%)
Ethnicity	
Hispanic or Latino	1 (5.5%)
Not Hispanic or Latino	16 (89%)
Unknown or do not wish to report	1 (5.5%)
Education	
Completed high school	2 (11%)
Some college or technical training	2 (11%)
University undergraduate degree	9 (50%)
University post graduate degree	5 (28%)
Marital status	
Single	10 (55.5%)
Married/partnered	7 (39%)
Divorced	1 (5.5%)
Employment status	
Working full-time	10 (55.5%)
Working part-time	2 (11%)
Working, but on medical leave	2 (11%)
Student	1 (5.5%)
Not working	3 (17%)
Cancer type	
Lymphoma	5 (28%)
Sarcoma or bone cancer	2 (11%)
Melanoma	2 (11%)
Breast	7 (39%)
Other (embryonal carcinoma and ovarian)	2 (11%)
Cancer stage (***n*** = 17)	
Stage I	5 (28%)
Stage II	4 (22%)
Stage III	2 (11%)
Metastatic	3 (17%)
Not applicable	3 (17%)
Cancer treatment type*	
Chemotherapy	14 (78%)
Surgery	13 (72%)
Radiation therapy	7 (39%)
Immunotherapy	8 (44%)
Hormone therapy	4 (22%)
Stem cell or bone marrow transplant	3 (17%)
Targeted therapy	2 (11%)
***Median*** (range) PROMIS scores at baseline	
Anxiety	59.55 (40.3–73)
Depression	51.97 (41–71.4)
Fatigue	54.2 (33.7–75.8)
Sleep disturbance	53.29 (32–65.9)
Physical function	48.1 (36.4–56.9)
Pain interference	52.25 (41.6–72)
Pain intensity	2.0 (0–4)
Ability to participate in roles and social activities	52 (27.5–64.2)
Self-Efficacy for Managing Chronic Conditions–Managing Symptoms	46.6 (35.7–63.5)
Digital Health Care Literacy Scale	
I can use applications/programs on my cell phone, computer, or another electronic device on my own (without asking help from someone else)	4.0 (3–4)
I can set up a video chat using my cell phone, computer, or another electronic device on my own (without asking for help from someone else)	4.0 (3–4)
I can solve or figure out how to solve basic technical issues on my own (without asking for help from someone else)	4.0 (3–4)
Months since last cancer treatment	
* Median (range)*	8.5 (1–18)

### Prior experiences with cancer treatment-related symptoms and AI use

Participants consistently described in the interviews a substantial gap between their symptom-related needs and the level of routine clinical care support available during and after cancer treatment. While survivors expressed appreciation for their oncology teams, many felt that time-limited visits constrained opportunities to discuss the breadth, severity, and persistence of treatment-related symptoms. Several participants noted that provider-delivered information regarding treatment side effects was often insufficiently comprehensive, particularly regarding long-term and late effects. As a result, many survivors felt underprepared for the physical and psychological challenges they encountered following treatment completion.

Participants reported a wide range of ongoing treatment-related symptoms affecting daily functioning and emotional well-being, including fatigue, pain, nausea, diarrhea, peripheral neuropathy, cognitive changes (“brain fog”), alopecia, insomnia, and anxiety. Fatigue and cognitive difficulties were frequently described as invisible or minimized symptoms, leading some survivors to feel that their concerns were not fully acknowledged in clinical encounters. These symptoms were often described as unpredictable, persistent, and disruptive to work, social roles, and mental health, extending well beyond the end of active treatment.

In response to unmet needs, many participants sought symptom-related information and validation outside of traditional healthcare settings. Online peer support groups, social media, and digital health resources (e.g., internet searches) were commonly used to normalize experiences, compare symptoms, and identify self-management strategies. While these platforms offered emotional support and immediacy, survivors also recognized variability in information quality and expressed uncertainty regarding the reliability of advice obtained through informal online channels.

Regarding prior experience with artificial intelligence, most participants reported familiarity with AI-powered tools (e.g., most commonly ChatGPT, as well as Microsoft Copilot, Google Pixel AI, and OpenEvidence) in everyday contexts, such as generating written content, organizing tasks, summarizing information, and travel planning. Several participants had also used digital assistants or search engines to obtain health-related information. This prior exposure appeared to contribute to openness toward AI-based symptom support, especially if designed specifically for healthcare contexts.

### Perspectives on generative AI chatbots for symptom support

Three key themes emerged from interviews with young adult cancer survivors regarding generative AI-powered chatbots for symptom support. Table 3 provides exemplar quotations supporting the themes. Additionally, Supplementary Information includes the framework matrix of participant responses to interview questions that support theme organization. The narrative below summarizes key findings and areas of convergence and variation across participants.

**Table 3:** Summary of themes and associated exemplar quotes from the semi-structured interviews with young adult cancer survivors

Themes	Exemplar quotes
The Need for Personalized, Emotionally Supportive, and Credible AI Symptom Guidance	“It’s important to know where the information comes from…if it’s generative AI from the internet, that’s risky; if it’s curated by professionals, it’s safer” (P7) “I would say, can the AI robot understand my situation or is it going to be more about everybody in general? I want it to be specific to me and how's the AI bot going to teach me specifically my disease, my experience, my relapses.” (P14) “To my knowledge, the chatbot is mostly for symptom tracking… but AYAs also need social-emotional support. It’d be nice if an AI chatbot could help with difficult conversations, family issues, and connecting to resources… not just the clinical side.” (P10)
Accessible and User-Centered Design Drives Engagement with AI Symptom Support	“I like apps because they're on my phone…just being able to open up an app and type or somehow interact, especially if you're feeling nervous or you just want somebody to talk to…It's nice to be able to do that from anywhere easily through your phone.” (P3) “If it used friendly, supportive, empathetic language like, Hi, how are you feeling today? or It’s completely normal to feel this way after treatment-that would be great. It validates my feelings. I’d love a chatbot that feels like a friend, uses simple, empathetic words, encourages me, and is non‑judgmental.” (P6)
Trust, Privacy, and Human Oversight as Preconditions for AI Symptom Support	“I think, my concern would be, if the chatbot kind of exaggerates the symptom I was describing, I would be concerned…So, either exaggerates or minimizes, that would be my concern.” (P5) “I think there's generally concerns about data privacy Many AI models aren’t transparent about how they use health information, and it’s unclear where the data goes or how it might be used in the future. That makes me a little hesitant.” (P9)

#### Theme 1: the need for personalized, emotionally supportive, and credible AI symptom guidance

Participants emphasized that personalization was central to the perceived usefulness of generative AI chatbots for symptom support. Survivors expressed frustration with generic or standardized advice that failed to account for individual treatment histories, symptom trajectories, and psychosocial contexts. Instead, they envisioned AI chatbots capable of integrating personal health information, such as cancer type, treatments received, time since treatment, and current emotional state to deliver tailored evidence-based guidance.

Beyond informational support, participants highlighted the potential psychosocial role of chatbots in addressing emotional distress and uncertainty. Survivors valued the idea of chatbots that could provide empathetic, validating responses using clear, nontechnical language. Regular check-ins and affirming messages were seen as mechanisms to reduce feelings of isolation and self-doubt, especially when symptoms were ambiguous or fluctuating.

While participants stated that they wanted to retain final authority over whether and when to seek medical care, many welcomed guidance to help interpret symptom severity and determine appropriate next steps. Chatbots were viewed as potentially useful in validating concerns, reinforcing symptom monitoring, and suggesting when contacting a healthcare provider might be warranted. Importantly, survivors distinguished between supportive guidance and autonomous clinical decision-making, emphasizing that chatbots should inform rather than direct care decisions.

Participants compared AI chatbots with online searches, which were often described as leading to information overload or exposure to unreliable sources. In contrast, survivors expressed trust in chatbots that were described as evidence-based, curated by healthcare professionals, and transparent about their sources. The ability to cite reputable references and explain the rationale behind recommendations was viewed as essential for credibility.

#### Theme 2: accessible and user-centered design drives engagement with AI symptom support

Accessibility emerged as a key determinant of engagement with AI-based symptom support. Although participants reiterated that healthcare providers remained their primary and most trusted source of care, many saw value in AI chatbots as a supplemental resource available outside of clinic hours. The 24/7 availability of chatbots was particularly appealing for managing symptoms that arose unpredictably or during periods of heightened anxiety.

Participants preferred chatbots with adaptable communication styles, emphasizing that language and tone should align with the purpose of the interaction. Professional, factual language was favored for symptom interpretation and clinical information, whereas a friendly, empathetic tone was valued for emotional support. Perspectives varied regarding preferred communication styles and features, with participants describing a desired balance between professionalism, warmth, and reassurance.

In addition, participants expressed preferences for flexible and user-friendly platforms, including mobile applications, web-based interfaces, patient portals, and multimodal input options such as voice or image uploads. Features that supported symptom tracking, visualization of symptom trends over time, and easy retrieval of symptom histories were perceived as particularly beneficial for self-management and preparation for clinical visits. Survivors appreciated the potential for chatbots to aggregate evidence-based resources and translate information into actionable recommendations, such as self-care strategies, identification of red-flag symptoms, and guidance regarding when to seek professional support. Several participants raised practical and system-level considerations related to cost, insurance coverage, and integration with existing healthcare systems. Perspectives varied regarding which implementation features would be most important for sustained use, although free or insurance-covered tools were generally perceived as more feasible for continued use.

#### Theme 3: trust, privacy, and human oversight as preconditions for AI symptom support

Despite general openness to AI-based symptom support, a substantial proportion of participants expressed concerns related to reliability, privacy, and data governance. Survivors questioned whether AI chatbots could accurately interpret symptom descriptions, particularly when symptoms were complex, subjective, or overlapping. Many supported the concept of symptom escalation to healthcare teams but strongly preferred manual control over when and how their information was shared. Automated data transmission without explicit user consent was viewed as undermining personal autonomy and potentially leading to unwanted clinical interventions. While most participants were comfortable sharing general symptom and treatment-related information, they expressed greater hesitation about sharing personally identifying data. Overall, participants articulated a clear boundary between supportive technology and autonomous clinical action. Generative AI chatbots were viewed as acceptable and potentially valuable tools for information, validation, and self-management support, but not as substitutes for clinical judgment or decision-making.

## Discussion

This qualitative descriptive study sought to explore young adult cancer survivors’ perceptions and experiences surrounding generative AI chatbots for symptom management. Participants conceptualized generative AI chatbots as tools to bridge gaps in survivorship care and not as a replacement for clinical care. Participants perceived AI as a means to supplement limited clinic time, provide real-time symptom guidance, and support self-management between visits. These findings align with emerging evidence that AI chatbot-enabled interventions can enhance patient engagement, improve symptom monitoring, and provide personalized guidance when clinical resources are constrained [7, 24]. Consistent with previous literature, a main perceived barrier to AI use for supportive care included concerns about privacy and confidentiality with regard to the data submitted to the chatbot [25].

Participants also reported the importance of addressing anxiety and broader psychosocial needs during survivorship. Consistent with existing AYA oncology literature, emotional distress, uncertainty, and unmet psychosocial needs remain common and are often insufficiently addressed during time-limited clinical interactions [26–28]. From participants’ perspectives, AI-based symptom support functioned not only as an informational tool but also as a source of emotional reassurance and continuity. Such emotionally responsive support may be particularly meaningful for young adults navigating ongoing symptoms alongside developmental, social, and vocational challenges.

Participants identified personalization as a key determinant of a chatbot’s perceived value, noting that standardized or generic responses often failed to reflect their treatment experiences, symptom trajectories, and broader psychosocial realities. This emphasis on tailoring aligns with existing digital health research demonstrating that personalized interventions are associated with greater engagement and relevance among individuals with cancer and other chronic conditions [29–31]. Participants also preferred professional, factual language for clinical symptom interpretation and empathetic, supportive language for emotional concerns. Growing evidence from oncology and other healthcare settings indicates that advanced conversational AI systems can deliver comprehensible and empathetic responses, underscoring the importance of chatbot designs that integrate factual accuracy with relational elements (e.g., empathy) [32–34]. Consistent with broader ethical perspectives, participants’ responses emphasized that AI should function as a supportive guide rather than an authoritative decision-maker, particularly in complex and subjective symptom contexts [35, 36].

Trust, transparency, and ethical integration were central to participants’ evaluations of AI chatbots. Survivors expressed concerns about potential misinformation, misinterpretation of symptoms, and inappropriate escalation. These concerns mirror growing ethical discussions in the literature regarding AI governance, privacy, and accountability in healthcare [37, 38]. Participants clearly differentiated healthcare-specific AI from general-purpose tools and emphasized the need for clinical validity, evidence-based content, and transparent safeguards. Together, these findings suggest that successful adoption hinges not only on usability but equally on credibility, accuracy, and contextual relevance.

Given the qualitative descriptive nature of this research, there are several different considerations for future research. First, future studies should evaluate AI chatbot interventions and assess clinical (e.g., feasibility and acceptability) and patient-reported outcomes among an AYA sample in a prospective, longitudinal study. Such research should also explore whether chatbot usage varies across physical and psychological symptoms and assess effects on symptom knowledge, self-management, and patient-reported outcomes. An important consideration in this type of future research is whether evidence-based symptom support should be integrated into widely used generative AI platforms or standalone oncology technologies. Existing platforms may offer greater accessibility and scalability but may generate inaccurate or unsupported information, underscoring the need for evidence-based content, transparency, and safety safeguards [39]. Standalone clinician-curated technologies may offer greater clinical oversight and safety but require appropriate training, implementation, and safeguards for privacy and patient autonomy [40]. Partnerships with clinical experts and professional organizations may help combine AI accessibility with clinically curated content. Initiatives such as the Multinational Association of Supportive Care in Cancer (MASCC) “Ask, Understand: Supportive Care in Cancer” platform illustrate this approach. The chatbot is free to use, draws responses from clinical practice guidelines and other scientific resources (e.g., webinars, assessment tools, and patient education) and does not retain participants’ responses [41]. Ultimately, if preliminary research demonstrates that AI chatbots are of value to AYA cancer survivors for supportive care concerns and help to improve quality of life like other digital health interventions for AYA cancer survivors [42], implementation research will be needed to identify scalable strategies for integrating AI tools into clinical workflows while ensuring patient engagement and protection of privacy.

### Limitations

This study has several limitations. The sample was small and homogenous with regard to sex, race, ethnicity and digital literacy, limiting generalizability to the broader AYA population. Also, given the rapidly evolving capabilities, availability, and public familiarity with generative AI, participants’ perceptions may only reflect the technological landscape at the time of data collection, and these perceptions may change as generative AI technologies continue to develop.

## Conclusion

Young adult cancer survivors expressed both optimism and caution regarding the integration of generative AI-powered chatbots into survivorship care. Features such as personalization, emotional support, and around-the-clock accessibility were viewed as central to perceived value, whereas concerns related to trust, privacy, data sharing, and information reliability were identified as important barriers to adoption. Participants perceived that thoughtfully designed and ethically implemented generative AI chatbots could serve as supportive adjuncts in survivorship care by facilitating symptom monitoring, supporting self-management, and providing timely psychosocial reassurance. Importantly, their potential lies not in replacing clinical care, but in augmenting existing healthcare relationships while respecting survivor autonomy and addressing the complex, multidimensional needs of young adult cancer survivors.

## Supplementary Information

Below is the link to the electronic supplementary material.

ESM 1 (XLSX 33.1 KB)

ESM 2 (DOCX 12.0 KB)

## Data Availability

The data that support the findings of this study are available from the corresponding author, RK, upon reasonable request.
