# Supplementary material for: Exploring young adult cancer survivors’ perspectives on generative AI chatbots for symptom support
Source: Support Care Cancer. 2026 Sep 23;34(10):1004. doi: 10.1007/s00520-026-11254-0 (PMC13597565; doi:10.1007/s00520-026-11254-0)
Supplement: Supplementary file 1 [file 520_2026_11254_MOESM2_ESM.docx]

**Supplementary Information**

**Framework Matrix of Participant Responses to Interview Guide Questions**

The framework matrix summarizes the analytic relationship among participant-level patterns in response to interview questions. Color coding was used to visually indicate the alignment of participant-level data with the overarching themes:

**Red** = Theme 1: The Need for Personalized, Emotionally Supportive, and Credible AI Symptom Guidance

**Green** = Theme 2: Accessible and User-Centered Design Drives Engagement with AI Symptom Support

**Purple** = Theme 3: Trust, Privacy, and Human Oversight as Preconditions for AI Symptom Support*.*

Uncolored text represents participant-level data that provides contextual or supporting evidence for the Results narrative but was not assigned to a specific overarching theme. Color coding is intended to facilitate transparency in linking the framework matrix to the Results narrative and does not represent frequency, weighting, or relative importance of codes.
